# An Edge-Fog Secure Self-Authenticable Data Transfer Protocol

**DOI:** 10.3390/s19163612

**Published:** 2019-08-19

**Authors:** Algimantas Venčkauskas, Nerijus Morkevicius, Vaidas Jukavičius, Robertas Damaševičius, Jevgenijus Toldinas, Šarūnas Grigaliūnas

**Affiliations:** Faculty of Informatics, Kaunas University of Technology, 51386 Kaunas, Lithuania

**Keywords:** fog computing, communication protocol, CoAP, information security, lightweight security protocols, wireless sensors, wireless actuators

## Abstract

Development of the Internet of Things (IoT) opens many new challenges. As IoT devices are getting smaller and smaller, the problems of so-called “constrained devices” arise. The traditional Internet protocols are not very well suited for constrained devices comprising localized network nodes with tens of devices primarily communicating with each other (e.g., various sensors in Body Area Network communicating with each other). These devices have very limited memory, processing, and power resources, so traditional security protocols and architectures also do not fit well. To address these challenges the Fog computing paradigm is used in which all constrained devices, or Edge nodes, primarily communicate only with less-constrained Fog node device, which collects all data, processes it and communicates with the outside world. We present a new lightweight secure self-authenticable transfer protocol (SSATP) for communications between Edge nodes and Fog nodes. The primary target of the proposed protocol is to use it as a secure transport for CoAP (Constrained Application Protocol) in place of UDP (User Datagram Protocol) and DTLS (Datagram Transport Layer Security), which are traditional choices in this scenario. SSATP uses modified header fields of standard UDP packets to transfer additional protocol handling and data flow management information as well as user data authentication information. The optional redundant data may be used to provide increased resistance to data losses when protocol is used in unreliable networks. The results of experiments presented in this paper show that SSATP is a better choice than UDP with DTLS in the cases, where the CoAP block transfer mode is used and/or in lossy networks.

## 1. Introduction

**Figure 1:**
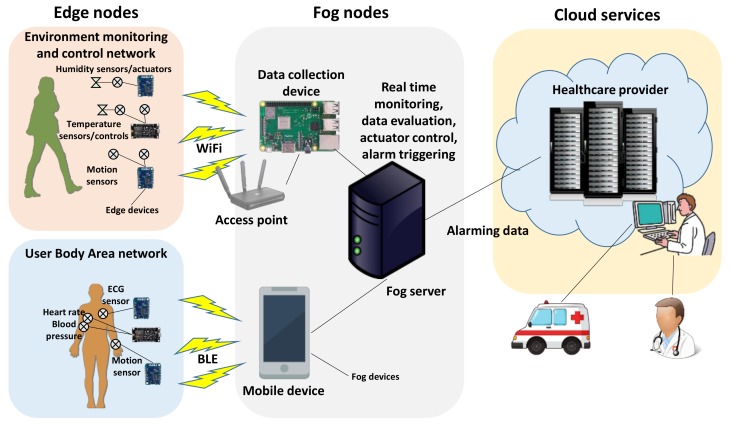
Three-layer Fog computing-based eHealth architecture.

The paradigm of Internet of Things (IoT) is used in many application domains, such as smart devices, smart homes, smart environment management, remote healthcare, etc. One of the main building blocks in these architectures are the networks of wireless smart sensors and actuators used to collect various data and send it to the data analysis and decision-making systems. The development of IoT introduces many new security challenges. As IoT devices are getting smaller and smaller, the problems of so-called “constrained devices” arise. We will discuss these challenges in the case of one of the fastest growing areas of IoT applications—healthcare, where measurement devices, such as wireless sensors, wearable devices, and mHealth apps, monitor patient biometric parameters and generate very large amounts of data that need to be processed effectively [1]. As a result, new distributed computing paradigms have emerged that combine portable devices with medical items over the Internet in order to be able to change remote telecommuting and services [2]. Use of Fog computing [3] architecture can reduce logistical requirements and related medical and hospital expenses [4]. Fog architecture could be used to reduce some limitations of WSNs [5] and help solve data routing [6] and data aggregation [7] challenges. Fog computing has many advantages and it is suitable for applications that are real-time critical, require quick response, and has low latency, particularly in the health care applications [8,9,10,11]. Fog computing-based eHealth architecture can be expressed as a three-layer hierarchical architecture with the Cloud-Fog-Edge devices (Figure 1). It provides a comprehensive solution from data collection, processing, and big data analysis to the Cloud platform.

The Edge nodes form the first layer of Fog architecture and they are various IoT-based smart devices (sensors and actuators) used to acquire data and control the environment. Various authors use the terms “End nodes”, “Edge nodes”, “End devices”, “User nodes”, “User devices”, or simply “Devices” to describe the first layer of the Fog architecture. In this paper, the terms “End nodes”, “Edge nodes” and “Edge devices” are used as synonyms. In healthcare applications, these devices form Wireless Medical Sensor Networks (MSNs), or Wireless Body Area Networks (WBANs) [12]. Often Edge devices are constrained [13], i.e., they have limited memory, CPU and/or energy resources, moreover, constrained wireless networks (lossy, with limited bandwidth, etc.) are often used. While modern constrained devices are able to implement and execute modern cryptographic primitives [14], the execution time is still significant, so less complex and more suitable lightweight communication protocols are often used to communicate with Edge nodes. Fog layer consists of various medium-power and medium-performance computing nodes, which collect data from Edge devices, process it and forward to the Cloud for further analysis.

Many modern security solutions are primarily intended to be used in traditional Internet and are designed to protect big enterprise networks, data centers, and some consumer products. These solutions are frequently based on the concepts of private networks, public networks, perimeter defense, etc. Traditional security methods and approaches could still be effectively used to protect the Fog node-Cloud part of the Fog architecture, while the Edge node-Fog node part requires a different approach. The Edge devices are constrained, use constrained wireless networks, and the environment is heterogeneous and distributed. Some wireless MSN generate a considerable amount of data, which are transferred from the Edge devices to the Fog nodes for further analysis. This data are mainly generated by the Edge devices, such as environment sensors, BAN sensors, portable devices, etc. [11,15]. The usual three fundamental components of security must be ensured: Confidentiality, data integrity, and device authentication [16], which may be challenging considering the limited resources of the Edge node devices. Therefore, more effective and requiring less computational resources security algorithms, as well as special lightweight communications protocols, such as CoAP (Constrained Application Protocol) [17] or MQTT (Message Queuing Telemetry Transport) [18], are usually used.

In this paper, we propose a novel lightweight secure self-authenticable transfer protocol (SSATP), which is intended to be used as transport protocol for CoAP for communications between Edge devices and Fog nodes. SSATP protocol uses modified UDP (User Datagram Protocol) packet’s headers to carry protocol handling and security information, lightweight symmetric encryption, nonces, secure hash functions and timestamps are used to provide user data confidentiality and to mutually authenticate Edge node devices to the Fog nodes, redundant data in the form of error correction codes are used to increase the resilience to network packet losses. The proposed protocol can successfully replace DTLS (Datagram Transport Layer Security), providing the equivalent security properties with a lower requirement for energy and computational power resources. The SSATP protocol ensures better transfer performance for applications where block-wise mode of CoAP protocol dominates, it provides better overall data delivery rate in unreliable networks with up to 10% of total packet loss, and consumes less energy when compared to plain UDP and DTLS working as transport protocols for CoAP in block data transfer mode. SSATP uses only symmetric cryptography primitives, does not rely on asymmetric ciphers, so it is easily implementable even on the smallest devices having low-end processing and memory capabilities. The paper is structured in the following way: The related work is presented in Section 2. We describe the proposed protocol in Section 3. The evaluation and experimental results are summarized in Section 4, and, finally, Section 5 is dedicated to conclusions and discussions.

## 2. Related Work

Dizdarević et al. [19] made a detailed review of the communications protocols in order to meet the communications requirements of IoT, and their suitability to implement the Fog-based IoT systems. They analyzed the application layer communication protocols (also called messaging protocols and machine-machine,) in the IoT architectures, taking into account the specific challenges of Fog and Cloud computing integration, including MQTT, AMQP (Advanced Message Queuing Protocol), DDS (Data Distribution Service), HTTP (Hypertext Transfer Protocol), XMPP (Extensible Messaging and Presence Protocol) and CoAP. At the transport level, these protocols use TCP or UDP. Security requirements are ensured using TLS or Datagram TLS (DTLS) [20] accordingly.

Quite a few works [21,22,23,24,25,26,27] studied various performance (bandwidth efficiency, power consumption, reliability) and security properties of these protocols. The suitability of lightweight CoAP and MQTT protocols to be used in highly constrained environments are analyzed in [28]. The research [28] shows that CoAP protocol used on top of UDP is more efficient than other analyzed protocols. Authors conclude that according to specific requirements for MSN listed above, the most efficient way to implement MSN is to use the CoAP protocol at the application layer and UDP at the transport layer [28].

The DTLS on top of UDP is used as the de facto standard security solution for CoAP protocol. DTLS is based on TLS with minimal changes caused by the nature of the underlying UDP protocol. As long as DTLS is only an adaptation of TLS protocol for use with unreliable transport and was never designed to consider all the special demands of IoT and constrained devices, the new versions of DTLS, which are more suitable for constrained environments, are emerging [29,30], and new lightweight authentication protocols are being proposed [31]. Despite these efforts, the optimization of DTLS for constrained environments is still an open issue [32,33].

The lightweight secure system for MSNs, proposed by He et al. [34], uses hash-chain based key update mechanism, as well as proxy protected signatures, to ensure secure data transfer and efficient data access control. This system is well fitted for use in constrained environments as it uses only symmetric ciphers and secure hash functions. Secure IoT-based healthcare system, introduced by Yeh [35], uses body sensor network architecture (BSN). The two main objectives of this system are to ensure efficiency and robustness of transmission using public IoT networks. To achieve confidentiality of the transmitted data and to ensure proper authentication of the smart devices, the local processing unit, and the BSN server, the system uses robust crypto-primitives and constructs two communication mechanisms. However, this solution ensures the security of communications only between users and smart devices.

A public-key-based lightweight authentication protocol for MSNs was presented by Hayajneh et al. in [36]. In this solution, all MSN nodes are divided into two classes: Sensors, which perform measurements of the human body, and actuators, which wait for directions from medical staff and perform corresponding actions. As the human health and wellbeing are involved here, the main security challenge is the authenticity of received command, as an incorrect invocation of some actuators may result in serious consequences. The authors show that confidential, tightly timed, and authenticated commands can be implemented using the proposed solution for MSN nodes.

Kraemer et al. [11] analyzed 163 papers and summarized the main requirements and issues for the Fog computing architecture: Low latency, energy efficiency, bandwidth requirements, etc. The special questions for Fog computing-based healthcare applications are also discussed and include bandwidth and latency requirements for various types of medical sensors, energy efficiency, security, interoperability, etc. Martínez-Pérez et al. [37] studied security and privacy problems in the area of mHealth. The authors focus their research in three areas: What is the current state of the legislation in the EU and USA related to the mHealth, what current academic literature is analyzing problems related to this topic, and, finally, the authors propose some recommendations, which are useful for designers and creators of mobile health applications, which have to satisfy the security, privacy and legislation requirements. Kotz et al. focus on the privacy and security challenges of mHealth technology [38]. Among other mHealth problems to be solved, the authors investigate the issues related to limited computational resources, network latency and throughput, dependability, privacy and security, and energy efficiency. Fog computing can help to address these challenges.

Stojmenovic et al. [39,40,41] investigated solutions to various Fog computing security challenges. The main security problems in the Edge device-Fog device layer are: Authentication of the devices, rogue nodes, confidentiality of data during network transfer, confidentiality and integrity of the stored data, secure computations in the Fog nodes, data privacy and intrusion detections and prevention [40,42]. Two different types of authentication problem may be distinguished in the Edge-Fog node part of Fog architecture while applied for healthcare applications [11,43]: Authentication of the Edge devices and authentication of the patient [44,45].

Xu et al. [46] suggested a privacy-preserving data integrity verification model for health cyber–physical system (CPS), which uses special data structures in lightweight streaming. Authors give a detailed description of the design, architecture of the solution, formal and security definitions, and specifications of communications protocol. The provided security and performance analysis results confirm the security and efficiency claims of the authors. Gope et al. [47] proposed a list of essential requirements for secure IoT based healthcare systems using BSN, which includes authentication, data privacy, anonymity, data integrity, data freshness, and secure localization. The authors then proposed an IoT-based healthcare system, called BSN-Care, which satisfies all security requirements. In our previous paper [48], we proposed a method for modification and evaluation of energy efficiency of SSL/TLS protocol, which achieves the best tradeoff between energy consumption and security of the transmitted data. Usman et al. [49] suggested authenticating data streams using a clustering-based technique. This method is energy-efficient, authenticates data stream and provides unaltered quality of service. Two-step authentication scheme is used: Node authentications and data authentication during secure transmission. The method uses crypto-hash tags, which organize all data packets into the chain. The usage of crypto-hash tags increases the amount of data transferred thought the network, which, in turn, increases the total energy consumption by about 20%. Wei et al. [49] used the multiple sink network structure with the Voronoi-based clustering algorithm to assign the optimal transmission range and power for each sink, and perform network clustering to effectively reduce the overall energy use of the network.

## 3. Lightweight Secure Self-Authenticable Data Transfer Protocol

Here, we propose a lightweight secure self-authenticable transfer protocol (SSATP) for Fog node-to-Edge node device communication in Fog computing. This protocol is intended for communications of constrained devices with limited energy and computation power using constrained networks, where packet loss is possible. The proposed protocol is intended to be used as transport protocol for CoAP, replacing the DTLS and UDP layers, and providing the security properties equivalent to that of DTLS. SSATP is based on ideas similar to our previous work [50], which are heavily modified to suit the new challenges provided by the request/response nature of the CoAP protocol. The new protocol is heavily adapted to closely meet the CoAP behavior.

The main advantage of SSATP over the standard UDP with DTLS is an increased resilience to data losses in unreliable networks and a more efficient way to transfer big data blocks using the CoAP protocol [51]. A short summary of its main properties is the following:

- Session-less communication with mutual authentication of server and client. No handshake or key agreement procedure is required to start the communication.
- Protocol provides confidentiality, integrity, and authentication of user data and increased resilience to data loss in network infrastructure.
- The length of authentication data and amount of redundant data, which are used to recover lost packets, is flexible and can be adjusted according to the requirements of a specific application.
- In the cases, where the resilience to data loss is not required, the protocol can provide zero data overhead, because all protocol and data flow management, as well as user data authentication information, are inserted into the headers of modified UDP packets, whereas the UDP packets’ data fields carry only useful user data.
- The protocol uses simple cryptographic primitives, such as symmetric ciphers, timestamps, nonces, and secure hash functions, which could be easily implemented in constrained Edge devices. No asymmetric encryption is used.

The implementation of the SSATP protocol is based on the following:

1. The protocol uses UDP packets with modified headers inspired by techniques used in covert channel solutions [52,53]. All protocol and data flow management information, as well as user data authentication information, are embedded in the header fields of UDP packets.
2. Edge device authenticators [54], timestamps and secure hash functions are used for mutual authentication of Edge node devices and Fog node devices.
3. Symmetric encryption algorithms and timestamping are used to provide user data confidentiality
4. Redundant data in the form of error correction codes (ECC) [55,56,57] and checksums [58] are added in the cases where a higher level of resilience to data packet loss during message transmission is required.

### 3.1. CoAP Protocol and Block Transfer Mode

**Figure 2:**
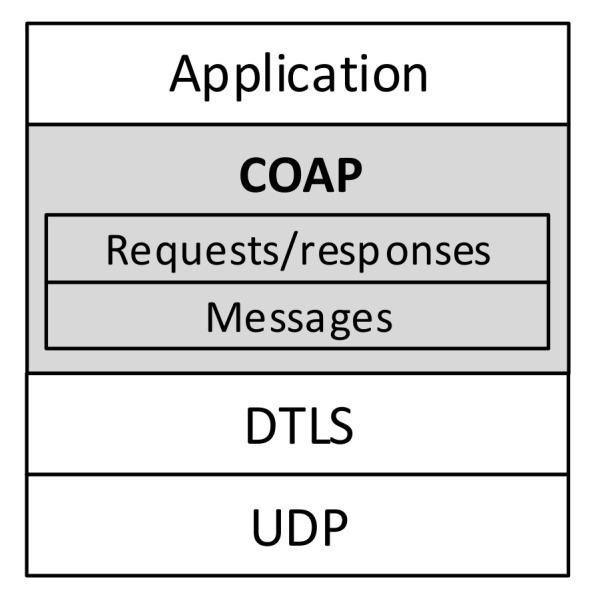
Datagram Transport Layer Security (DTLS)-secured Constrained Application Protocol (CoAP) architecture.

Constrained application protocol [17] is an HTTP-like request/response data transfer protocol primarily used for communication with constrained devices and/or using constrained networks. CoAP uses UDP as transport protocol and DTLS in the scenarios, when secure communications are required (Figure 2).

CoAP works well when small payloads are used in reliable networks and packets are small, and do not need fragmentation and resequencing. Transports used with CoAP (UDP and DTLS) provide only the service of packet fragmentation, which is not trivial to implement in constrained devices, therefore, many practical implementations try to avoid this by limiting data size transferred using one request/response pair. The authors of the CoAP standard allow developers to avoid IP fragmentation by using Block options in the CoAP protocol, for transferring large data payloads [51]. However, the main disadvantage of block transfer is that information is transferred in small blocks using many request/response pairs, which causes a large drop in an average data transfer speed. The situation is even worse, when CoAP is used in unreliable networks. In this case, in the event of the packet drop, the packet receiving party must wait for timeout and send retransmit request.

These shortcomings may be partially solved by using the proposed SSATP protocol as a transport for CoAP in place of UDP with DTLS.

### 3.2. Modified Data Transfer Protocol for CoAP

We propose to use the SSATP protocol as a secure transport protocol for CoAP. In SSATP, all additional transfer flow handling information and user data authentication codes are embedded in the headers of modified UDP packets. The structure of the standard UDP packet is unchanged, only some header fields are used differently. The communicating devices are authenticated using authenticators, which are generated from the secure device identifiers (sids) and timestamps using secure hash functions. The authentication data are processed and embedded into modified UDP headers. UDP is unreliable and does not guarantee packet delivery, preservation of original order, or deduplication of packets, therefore all data packets must carry packet order information. ECC are used for restoring data if some packets are lost during the transfer.

Authentication of the sender device and user data integrity is ensured by using the CCM mode for data encryption [59], which is also one of the mandatory-to-implement encryption modes of DTLS specified in the CoAP protocol standard. After the encryption of the data, the authentication field is generated automatically. During data encryption, the sending side adds a secure device identifier and timestamp information as additional authenticated data and nonce as parameters for CCM encryption mode. Based on the preferred length of the authentication field produced by CCM encryption and the size of the data provided by the higher levels of network stack (CoAP protocol), the data are divided into several packets grouped into the same segment $s_{i}$. Each segment is assigned a sequence number i=0, 1, …, Each communicating party counts the segments sequentially and independently. The optional packet with error correction data covering data and authentication field may be added to the segment as the last packet in the segment. The total length of the segments may vary and is provided to the receiving party in the special field in modified UDP packet header. All data packets $p_{i,j}$ comprising the same data segment $s_{i}$ are ordered and have the sequence number j, j=0, 1, …, n−1 assigned. The segment number i, segment size n , and packet number j are transmitted with each data packet as a part of the modified UDP header. The segment number, segment size and packet numbers are used by the receiver to reorder packets, when the packets are received out of their original order.

**Figure 3:**
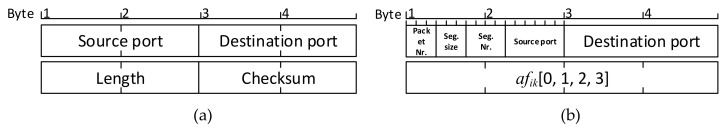
Proposed modifications to the User Datagram Protocol (UDP) packet’s header. Standard UDP header (**a**) and modified header (**b**).

The proposed modification of the UDP packet’s header fields is presented in Figure 3. The destination port field is left untouched from the original UDP packet header [52]. The UDP data length and checksum fields are used to store the k -th fragment (four bytes in length) of user data authentication field $af_{i,k},\;k=0,\;1,\;\ldots ,\;n-1$. The source port field is divided into four parts. The first three bits are used to store the sequential number of the packet in the current segment of data (j), the following three bits are used to identify the total amount of packets in the current segment (n), and the next four bits are used to identify the number of the current data segment (i). Due to the fact that the CoAP protocol is of request/response type of protocol, the source port number of each packet must be preserved. For this purpose, the remaining six bits are used to encode the source port. The reconstructed source port reported to the upper levels of network stack is calculated by adding the remaining six bits from the modified UDP header to the reserved CoAP port value 5683, thus ensuring the possible range of source ports of 5683–5746.

For example, if the AES_CCM encryption algorithm with eight bytes of authentication field is used and redundancy option is enabled, then the length of the according data segment is n=2+1 data packets, because eight bytes of authentication information fit into two packets’ headers with an additional one packet used for checksums of data and authentication field.

The particular selection of the UDP header fields was reasoned following these assumptions:

1. The length of the UDP data is not very important and redundant, as the data length could be easily calculated using the IP header;
2. Although the checksum field is not compulsory in UDP header, data integrity is checked in the data link layer. Additional authentication and data integrity checking are provided by SSATP protocol.

The modification of the UDP header fields sometimes may cause issues in traditional IP networks including routers, firewalls, etc. This is not a problem as long as SSATP protocol is used for communications between the Fog and Edge node devices, where only the OSI Level 2 network devices are dominating. Our inspections during the experimental testing of the protocol show that all modifications do not cause any additional problems in operating system network stack as long as we use only low-level network libraries (e.g., libpcap [60], winpcap [61], etc.).

Modification of UDP fields causes incompatibility of the proposed protocol with standard UDP-based implementations of CoAP. As a consequence, it is necessary to implement and use the SSATP protocol on both communicating devices.

### 3.3. Generation of Secure Device Identifiers and Registration of Edge Devices

The first step while organizing a new Fog node and adding a new Edge device to the infrastructure is the registration. Registration starts with the generation of the sid for a new Edge device, which is secret and known only to the Edge device and to one of the Fog nodes. As long as the CoAP protocol is request/response protocol, the new Edge device also has to know ***sid*** of the Fog node it intends to communicate with. The secure device identifier is transmitted to the Fog node using a secure communication channel and is stored in the Fog node. An initial secure channel must be used to generate and transfer secure sids to the appropriate devices. Secure channel can be established with the direct wire connection using USB or UART, with wireless communication using factory one-time-password or in other manner.

Data authentication information (encryption keys, additional authentication data, and nonce used in CCM cipher mode) is created from the secure device identifier of the sending and receiving parties, so these identifiers must be unclonable, truly random, contain enough entropy, have sufficient length, and are not stored on the device. For generation of sids, physical unclonable functions (PUFs) [62] are used. In the scenarios where special hardware is not available, a software-based secret encryption key generation algorithm, as the one proposed and evaluated in our previous work [54], may be involved.

### 3.4. Encryption Parameters’ Generation, Authentication and Redundant Data Calculation

All the packets of the SSATP protocol include essential data flow management information, i.e., segment number, current segment size, and packet sequence number in the current segment. These numbers are calculated independently on the server and client sides and are used on the receiving party to check segments’ authenticity and to restore packet order and/or lost packets. SSATP uses a symmetric cipher in the CCM mode. This modification of the symmetric cipher provides encryption and data authentication and requires the following parameters: The encryption key (e.g., 128 bits, if standard AES is used), additional authenticated data, and nonce (7–13 bytes).

1. New encryption key $ek_{i}$ is generated for each new segment of data. $ek_{i}=H(sid_{s}\;||sid_{d}\;\left|\left|\;ts\;\right|\right|\;i)$, where $sid_{s}$ is the secure identifier of the source device, $sid_{d}$ is the secure identifier of the destination device, ts is the current timestamp, i is the sequential number of segment and *H* is the secure hash function. When the result is too long for the key of the encryption algorithm, it is truncated.
2. Additional authenticated data $ad_{i}$ is a simple concatenation of all information available in the headers of the packets: $ad_{i}=n\left|\left|\;i\right|\left|port_{s}\right|\right|port_{d}$, where *n* is the size of the current segment, *i* is the sequence number of the current segment, $port_{s}$ and $port_{d}$ are source and destination ports of the packets.
3. Nonce value is also unique for each new segment of data: nonce= H(ts || i), where ts is exactly the same timestamp value as used in key generation, and *i* is the sequence number of the current segment. Nonce value is then truncated to first eight bytes.

On the packet sending side, the user data are encrypted using a symmetric cipher in the CCM mode and the calculated values of encryption parameters. The result of encryption is ciphertext $ct_{i}$ (with a size equal to plaintext) appended with authentication field $af_{i}$. The size of the authentication field may vary according to the CCM standard from 4 to 16 bytes. The authentication field is then separated from ciphertext and divided into *m* parts $af_{i,k}$, k=0,1,…,m−1, where $m=lenght\left(af_{i}\right)/4$. Parts of the authentication data are then inserted into headers of the corresponding packets. Ciphertext is also divided into *m* parts $ct_{i,k}$ and each part is used as a payload of the corresponding packet.

If optional redundancy information is required, then chosen error correction function *fecc* is used, and ECCs are calculated for all parts of authentication fields $af_{i,m}=ecc_{auth}=fecc\left(af_{i,0},\ldots ,af_{i,m-1}\right)$ and ciphertexts $ct_{i,m}=\;ecc_{ct}=fecc\left(ct_{i,0},\ldots ,ct_{i,m-1}\right)$. The additional packet is added to the current data segment with $af_{i,m}$ in the header and $ct_{i,m}$ as a payload.

Finally, all packets involved in the segment are sent to the receiver.

To authenticate the source of newly received data, the receiver must collect all packets of the current segment i, concatenate all payloads $ct_{i,k}$, k=0, 1,…,m−1 into ciphertext $ct_{i}$, extract the authentication data $af_{i,k}$, k=0, 1,…,m−1 combine them and append to $ct_{i}$. The CCM mode of a symmetric cipher automatically decrypts and authenticates the data.

If some of the packets go missing during transmission, and redundancy data packet is available, then the lost fragment is restored using the ECC before the decryption takes place.

## 4. Evaluation of the Protocol

### 4.1. Qualitative Comparison

The proposed SSATP protocol has an advantage over DTLS in the simplicity of the procedure of the registration of a new device to the Fog node. Only the lightweight cryptographic functions (symmetric cipher and hash function) are required to authenticate devices and provide confidentiality and authenticity for the transferred data. On the other hand, the DTLS protocol often requires asymmetric ciphers, generation, and distribution of key pairs to all devices involved in the exchange of information. Moreover, the verification process of key pairs provided by other communicating party is not a trivial task for the resource-constrained devices.

The main disadvantage of the SSL/TLS-based protocols in the constrained devices and networks is the handshake procedure, which requires considerable processing resources and reliable network connection. Even slight data loss in the network causes significant delays, when one side is waiting for a missing packet, and after the timeout tries to retransmit the last request. If the handshake happens frequently, it can significantly increase network traffic and cause delays. The SSATP protocol does not require any special negotiation stage to set up the connection between the server and the client and, consequently, does not have such shortcomings. Moreover, the DTLS protocol adds authentication and data flow control information to the user data causing a slight increase of network traffic. The SSATP protocol uses only not essential UDP header fields to store all additional information, therefore, data fields of the network packets are untouched, thus ensuring zero traffic overhead.

The DTLS protocol has to use a new handshake procedure, when the connection between communicating parties is resumed after a long delay, but the proposed SSATP protocol can resume the sessions almost instantly as authentication of source is possible after one full segment of data is collected and decrypted.

The DTLS protocol is not specially targeted to be used under non-ideal network conditions and it does not have any special means to deal with data loss or changes of the packet order. Only the handshake stage of DTLS requires correct data delivery and, in the case of packet loss, the timeout and packet retransmission techniques are used. The proposed SSATP protocol uses ECC for integrity check of protocol and used data. Moreover, the ECC data could be also used to restore the entire data segment, if one packet is missing, thus providing higher resistance to data loss when using network infrastructure with possible packet losses.

One side effect of usage of multiple smaller packets is increased efficiency of the CoAP protocol in the block transfer mode. The CoAP protocol may use logical packets bigger than maximum transmission unit (MTU) of the network, considering that SSATP will divide them into a few smaller packets, thus ensuring that the final size of the packets on the media will be less than MTU and will not cause packet fragmentation. This property allows to use fewer request/response exchanges and speed up data transfer in the CoAP block transfer mode.

One of the negative sides of the proposed SSATP protocol is the need to use multiple smaller packets instead of one bigger packet in case of plain UDP or DTLS. The number of packets increases as longer authentication fields are used in the CCM encryption mode. In practice, this impact is not very critical as all multiple packets comprising one segment of data are sent in one take without waiting for the response from the other side. Our practical experiments show that the impact of sending three or five smaller packets instead of one bigger packet is not very significant when compared with delays of one request/response exchange. Moreover, the receiving party is aware of the timing of the packets from the same segment and could use significantly smaller timeout values while communicating in lossy networks.

### 4.2. Security Assessment

The proposed protocol is able to provide several security properties, which are required in various application areas, e.g., MSNs, WBANs, etc. Confidentiality of the data is preserved using symmetric encryption, and it is uncompromised as long as the sids of the communicating parties are kept secret. The integrity of the data is ensured by using the CCM encryption mode and the authentication data field of sufficient length. Communicating devices are authenticated to each other so the authenticity of the data source is ensured. The protocol does not provide means for authentications of the patient, so this property must be ensured by additional means (if required by a particular application). The protocol ensures the data freshness property, as timestamps are used on both sending and receiving parties. Moreover, the replay attack is not possible due to timestamps and counters used to encrypt the data. The impersonation attack is not possible if the sids of the devices are not compromised. The protocol provides increased data availability as it uses redundant data, which could be used to reconstruct the missing fragments of the data. SSATP does not have the property of forward secrecy, because after the compromisation of the sids belonging to both communicating parties, all the data collected in the past could be decrypted, if the exact time of the collection is also available. The protocol is resistant to the man-in-the-middle attack.

### 4.3. Performance Comparison

To assess the performance of the proposed protocol, a CoAP request making client and different length responses providing server were created. Raspberry Pi embedded computer (Model B rev. 2, BCM2835 CPU, 512 MB RAM) with “Raspbian GNU/Linux 9 (stretch)” was used as the CoAP server. Performance was measured at the client device, which was a standard PC running Windows 10 operating system. All tests were performed in the following fashion: (1) The client computer sends a short GET request to the server (the length of the expected response is indicated in the request’s parameters), (2) the server generates response of the required length and sends it to the client, 3) the client has to wait for the response and then sends a new request.

The SSATP protocol was implemented in Java programming language using security libraries from Bouncy Castle [63]. The low-level access to the UDP packets’ headers was provided by the jnetpcap Java library [64] acting as an interface to the low-level libpcap and winpcap system libraries. The Eclipse Californium [65] library was used to implement the CoAP server and client. The Scandium [66] library was used for tests involving DTLS.

Two modifications of the SSATP protocol were implemented. The first implementation (labeled as “M64”) used segments of two user data packets and one redundancy packet. The AES block cipher in CCM mode producing eight bytes of authentication data was used. To calculate the checksum values, a simple XOR function was employed. The headers of the first two packets of the segment carried an eight-byte authentication data, and the last packet carried the checksums of the first two parts of the digest and encrypted user data.

The second implementation (labeled as “M128”) used AES block cipher in the CCM mode producing 16 bytes of authentication data. All user data was split into payloads of four packets and the authentication data was embedded into the corresponding headers. The fifth’s packet’s payload was used for the ECC values of the first four data packets. To calculate checksums, we used the XOR function.

The performance characteristics of the proposed method were evaluated using a simple CoAP server and client with four different underlying transport protocols. (1) Standard UDP transport protocol, which does not ensure source authentication or confidentiality was used to evaluate baseline performance of the test setup, (2) DTLS over standard UDP was used as standard secure mechanism for CoAP, DTLS used the TLS_ECDHE_ECDSA_WITH_AES_128_CCM_8 ciphersuite, which provides the same level of security as M64 version of SSATP, (3) SSATP protocol implementation M64 using AES in CCM mode and producing eight bytes (64 bits) of authentication information, (4) M128 modification of SSATP using AES in CCM mode and producing 16 bytes of authentication data.

### 4.4. Experimental Results

During the experimental evaluation of the performance, the client sent requests for a specific length of data and waited for the corresponding response from the server. After the response was received, the client sent a request for another bit of server data. The procedure was repeated until the total amount of 1 MB of server data was received by the client. E.g., in the case where 128 B size data chunks were used, the total amount of 8192 request/response actions, each carrying 128 B of server data were performed.

**Figure 4:**
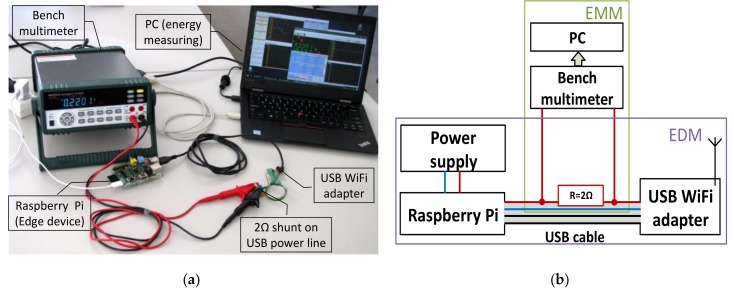
Energy consumption measurement setup: (**a**) Overall picture of the setup, (**b**) principal diagram of the setup, here EDM—Edge device module, EMM—energy measuring module [50].

The energy consumption on the CoAP server device’s network adapter was measured to compare the power efficiency of the proposed method with the standard transport protocols. We measured only the energy drained by the USB Wi-Fi adapter (Digitus Wireless 150N USB adapter) attached to the Raspberry Pi computer. The arrangement used for energy measurement is summarized in Figure 4

Energy consumption was measured at the server network interface card, by cutting USB cable, inserting current shunt and using a bench multimeter (The Mastech MS8050 Benchtop Multimeter) connected to the PC to record current consumption during data transmission. We used Matlab (The MathWorks Inc.: Natick, MA, USA) software to process collected data, to calculate average quiescent current and the total energy consumption during data transmission. DTLS and M64 protocols were using the same encryption algorithms, this guarantees that power consumed for data encryption should be the same by these two protocols.

**Figure 5:**
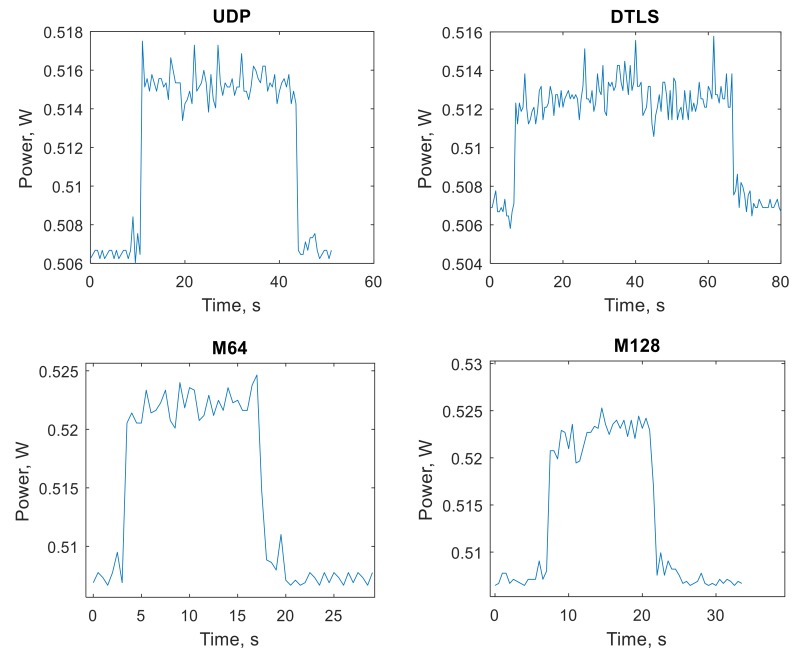
Power consumption of the Wi-Fi network card while transferring 1 MB of data using 2048 B data packets and different transport protocols.

Figure 5 shows the power consumption of the Wi-Fi network interface card while transferring 1 MB of data using 2048 B data packets and different transport protocols.

**Figure 6:**
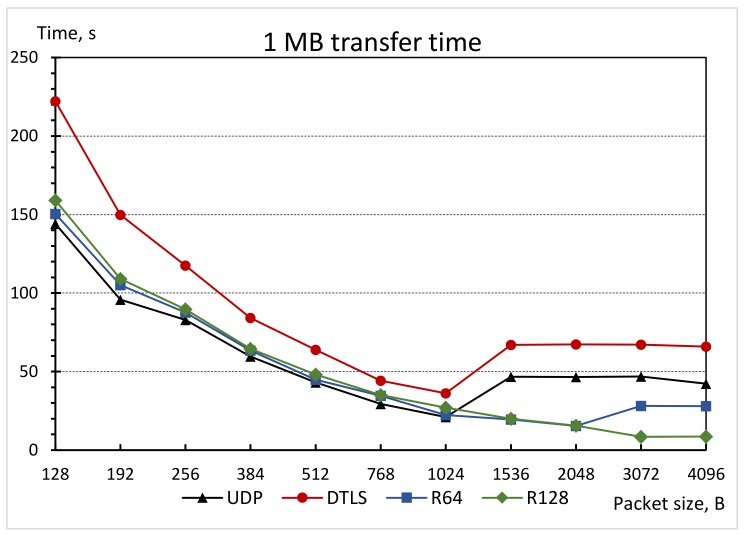
Comparison of time needed to transfer 1 MB of data using different transport protocols.

Figure 6 presents the performance of the CoAP protocol using four aforementioned transport protocols with regard to transmission time while transmitting 1 MB of user data (from server to client only, additional traffic required for all requests and protocol information are not counted here) using different CoAP protocol level user data sizes. The plain UDP achieved the best result, but both implementations of the SSATP method are very close competitors, even considering the fact that they are providing encryption and data authentication. The standard DTLS transport is the worst performer. This is mainly caused by quite significant time used by handshake procedure (performed only once at the very beginning of each data transfer session) and probably due to the additional computations performed on the sending device.

The main criterion for selecting the parameters of Californium CoAP library was that the maximum size of any packet on the network media should not exceed 1024 bytes (excluding the overhead data introduced by the whole network protocol stack starting with the CoAP itself). The Californium library parameters used in this experiment were the following: MAX_MESSAGE_SIZE = 1024 (default), PREFERRED_BLOCK_SIZE = 512 (default), MAX_RESOURCE_BODY_SIZE = 8192. Only the first parameter was doubled for M64 protocol and quadrupled for M128 protocol, as these transport protocols divide CoAP messages into smaller network packets by themselves. The final result of network packet size and transfer mode used depending on the size of the user data transferred using CoAP protocol is summarized in Table 1.

The performance evaluation results clearly show that when using user data sizes above 1024 B, the CoAP block transfer mode kicks in and causes a significant decrease in overall transfer performance. The only difference here is that M64 and M128 transport protocols allow to start using block transfer mode starting from bigger user data sizes (2048 bytes and 4096 bytes, respectively).

**Figure 7:**
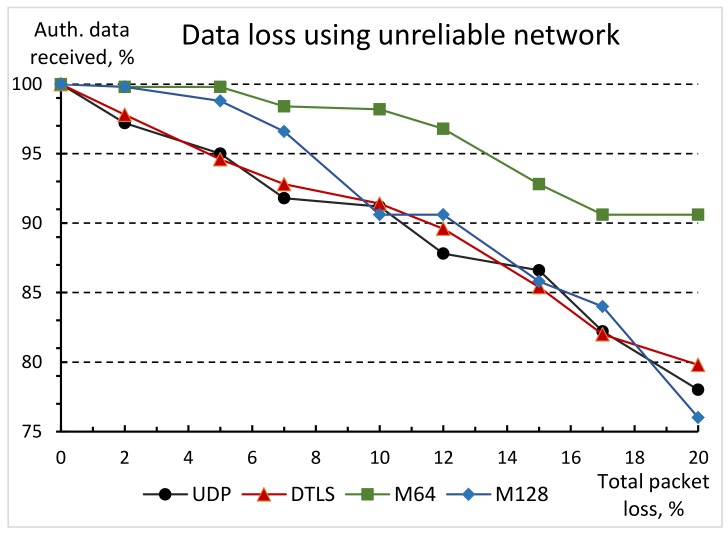
Comparison of user data losses using different transport protocols in lossy network.

We used the NetEM [67] (Network Emulation) tool to assess the performance of the SSATP protocol’s modifications in lossy network conditions. NetEM was used on the CoAP server side (the Raspberry Pi) to emulate random network packet loss. The receiver collected all data packets and restored missing data packets (if any). The results are shown in Figure 7.

During this experiment, the non-confirmable CoAP messages were used to transfer 1 MB of user data in 1024 B chunks, so block transfer mode was not engaged in all cases of different transport protocols. Results show that both implementations of the SSATP protocol allow to achieve less overall data loss in non-ideal network settings by using the redundant data packets.

The M128 modification is a better choice in poor quality networks only if average network data loss does not exceed 10%. Starting from 15% of network loss, M128 protocol shows even worse results than plain UDP and DTLS. For example, in networks with average 25% of data packets loss, M128 delivers only 64.6% of data packets, while UDP and DTLS settle around 75%. The M64 implementation tells a different story, even in the case of 40% of average data loss in the network infrastructure, the M64 manages to get significantly better final reliability compared to the plain UDP or DTLS (66.2% vs. 60% respectively).

**Figure 8:**
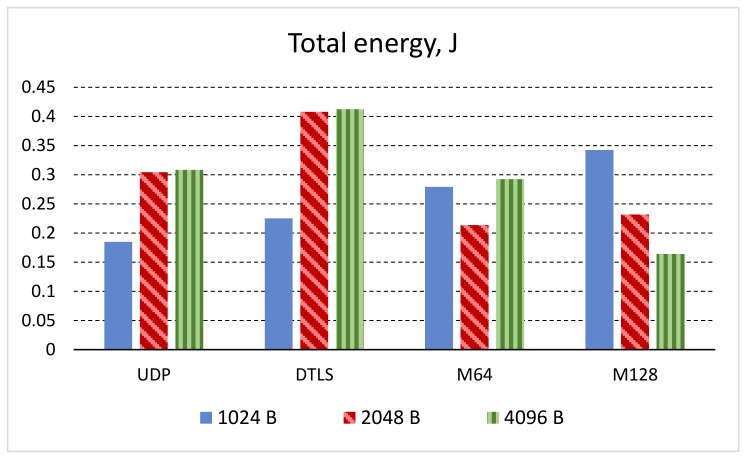
Comparison of energy consumption.

To summarize the differences of energy requirements induced by different transport protocols, we used all four protocols with user data sizes of 1024B, 2048B, and 4096B to transfer 1 MB of useful data from the server to the client. Moreover, the quiescent energy used to power the USB Wi-Fi adapter (which is significant, as seen from Figure 5) was not included in this evaluation, thus leaving only the energy used for the transmission of the data. The results are summarized in Figure 8.

From the results, we can clearly see that M64 and M128 protocols use more energy in the case of data packets of 1024 bytes. This is because in all four cases, CoAP is not using block transfer mode, but the M64 and M128 transport protocols add additional redundancy data packet to each data segment and uses a higher count of smaller data packets to transfer the same amount of user data. The situation changes, if the 2048 B and 4096 B data packets are used. In these cases, M64 and M128 benefits from avoiding block-wise transfer mode, thus causing fewer request/response exchanges on the CoAP level, and consequently, significantly decreasing the total energy required to transfer the same amount of user data.

## 5. Conclusions and Future Work

A new lightweight secure self-authenticable transfer protocol (SSATP) was proposed in this paper. This protocol is intended to be used as a transport protocol for CoAP in constrained environments, primarily for Fog computing communications between Edge nodes and Fog nodes. The SSATP protocol is well suited for applications where block-wise transfer of CoAP protocol dominates and/or network infrastructure is lossy.

SSATP is a lightweight secure communications protocol, which provides authentication and confidentiality to the user data. It uses a covert-channel-inspired method to embed data flow management and security data in modified UDP packets’ header fields. The protocol provides security properties comparable to DTLS and does not use handshake procedure or asymmetric ciphers. It is well suited for constrained devices as it uses only symmetric ciphers and hash functions.

Two modifications of the SSATP protocol were implemented and experimentally compared with plain UDP and DTLS used as transports for the CoAP protocol. The analysis of the results shows that the proposed modifications provide very similar performance characteristics when compared to the plain UDP and are marginally faster when compared to DTLS. However, in the cases where the CoAP protocol starts to use the block-wise transfer mode, both SSATP protocol implementations are significantly faster than UDP, even when considering that SSATP additionally provides authentication and confidentiality for the user data.

In network environments, where significant data loss is expected, SSATP uses an additional redundant data packet to provide increased data transfer reliability. The experimental results show that both modifications are preferable to plain UDP and DTLS in network environments, where an average data loss is up to 10%. On the other hand, the M64 modification provides significantly better data transfer reliability in the networks with up to average 20% packet drop.

Our energy consumption experiments show that both modifications of the SSATP protocol consume slightly more energy when CoAP protocol is used in its standard mode. This is the consequence of the fact that both modifications use additional packet with redundant information. However, the picture changes when the CoAP block-wise mode is used. In this case, both modifications of the SSATP protocol consume less energy when compared to plain UDP and DTLS.

A number of interesting aspects of the proposed protocol could be investigated in the future. It is unclear how the SSATP will perform in the real-life sensor networks with many Edge devices all trying to communicate with their respective Fog nodes. It would also be interesting to see the power consumption differences on the Edge devices themselves while executing different protocols, and to compare the performance of the protocol on devices with different processing power.

The new proposed CoAP standard modifications [68] specify the requirements to use CoAP over TCP and TLS. In the future, we will compare the performance of these implementations.

Our final conclusion and recommendation is that the SSATP protocol should be used as transport for the CoAP protocol in the situations where network infrastructure is unreliable and/or large user data blocks are frequently transferred using the CoAP protocol’s block-wise mode.

## Figures and Tables

**Table 1:** Data transfer mode used in various cases of transport protocol and user data sizes.

User Data Size, B	UDP, DTLS		M64		M128	
	Transfer Mode	Max. Packet Length on Media, B	Transfer Mode	Max. Packet Length on Media, B	Transfer Mode	Max. Packet Length on Media, B
≤1024	Plain packet	1024	Plain 3 packets	512	Plain 5 packets	256
1025–2048	Block-Wise transfer	512	Plain 3 packets	1024	Plain 5 packets	512
2049–4096	Block-Wise transfer	512	Block-Wise transfer (3 packets per block)	512	Plain 5 packets	1024
>4096	Block-Wise transfer	512	Block-Wise transfer (3 packets per block)	512	Block-Wise transfer (5 packets per block)	512
